# An Evaluation of the Stemness, Paracrine, and Tumorigenic Characteristics of Highly Expanded, Minimally Passaged Adipose-Derived Stem Cells

**DOI:** 10.1371/journal.pone.0162332

**Published:** 2016-09-15

**Authors:** Oula El Atat, Diane Antonios, George Hilal, Nabil Hokayem, Joelle Abou-Ghoch, Hussein Hashim, Rim Serhal, Clara Hebbo, Mayssam Moussa, Nada Alaaeddine

**Affiliations:** 1 Regenerative Medicine and Inflammation Laboratory, Faculty of Medicine, St. Joseph University, Beirut, Lebanon; 2 Toxicology Laboratory, Faculty of Pharmacy, St. Joseph University, Beirut, Lebanon; 3 Cancer and Metabolism Laboratory, Faculty of Medicine, St. Joseph University, Beirut, Lebanon; 4 Department of Plastic& Reconstructive Surgery, Hotel Dieu de France, and Faculty of Medicine St Joseph University, Beirut, Lebanon; 5 Medical Genetics Unit, Faculty of Medicine, St. Joseph University, Beirut, Lebanon; 6 Department of Plastic& Reconstructive Surgery, Fuad Khoury Hospital, Beirut, Lebanon; Instituto Butantan, BRAZIL

## Abstract

The use of adipose-derived stem cells (ADSC) in regenerative medicine is rising due to their plasticity, capacity of differentiation and paracrine and trophic effects. Despite the large number of cells obtained from adipose tissue, it is usually not enough for therapeutic purposes for many diseases or cosmetic procedures. Thus, there is the need for culturing and expanding cells in-vitro for several weeks remain. Our aim is to investigate if long- term proliferation with minimal passaging will affect the stemness, paracrine secretions and carcinogenesis markers of ADSC. The immunophenotypic properties and aldehyde dehydrogenase (ALDH) activity of the initial stromal vascular fraction (SVF) and serially passaged ADSC were observed by flow cytometry. In parallel, the telomerase activity and the relative expression of oncogenes and tumor suppressor genes were assessed by q-PCR. We also assessed the cytokine secretion profile of passaged ADSC by an ELISA. The expanded ADSC retain their morphological and phenotypical characteristics. These cells maintained in culture for up to 12 weeks until P4, possessed stable telomerase and ALDH activity, without having a TP53 mutation. Furthermore, the relative expression levels of TP53, RB, and MDM2 were not affected while the relative expression of c-Myc decreased significantly. Finally, the levels of the secretions of PGE_2_, STC1, and TIMP2 were not affected but the levels of IL-6, VEGF, and TIMP 1 significantly decreased at P2. Our results suggest that the expansion of passaged ADSC does not affect the differentiation capacity of stem cells and does not confer a cancerous state or capacity in vitro to the cells.

## Introduction

Regenerative medicine is an exciting new field in which different techniques are used to mend damaged organs and tissues. Adult mesenchymal stem cells represent an attractive candidate for tissue regeneration and repair because they have low immunogenicity, are non-tumorigenic and are not subject to any ethical issues.

The International Society of Cellular Therapy (ISCT) proposed the minimum criteria for defining human mesenchymal stem cells (MSCs). First, these cells are plastic adherent and have a fibroblast-like morphology. Second, they must express CD73, CD90, and CD105, but lack the expression of CD34, CD45, CD14 or CD11b, CD79α or CD19, class II major histocompatibility complex (MHCII) molecule (mainly HLA-DR) and co-stimulatory molecules such as B7-1, B7-2, CD80, CD86, CD40 and CD40L. Third, they must be able to differentiate in vitro into mesodermal cellular lineages, more specifically adipocytes, osteoblasts, and chondrocytes [1–4].

MSCs were first isolated from bone marrow by Friedenstein *et al*. in 1976 [5, 6], and then named and characterized by Caplan in 1990 [7–9].

In 2001, Zuk *et al*. identified and characterized an alternative method to isolate mesenchymal stem cells from adipose tissue instead of bone marrow; they were subsequently known as adipose-derived stem cells (ADSCs) [10]. ADSCs comprise 2% of the nucleated cells in lipoaspirate which contains stromal vascular fractions (SVFs). SVF comprise pre-adipocytes, vascular smooth muscle cells, fibroblasts, resident monocytes/macrophages, endothelial cells, lymphocytes and is composed mainly of fat cells organized into lobules [10, 11]. The techniques used to isolate bone marrow-derived stem cells are considered invasive and painful and are associated with potential donor site morbidity and high contamination risks [12]. Adipose-derived stem cells are considered a more advantageous cell source than bone marrow-derived stem cells because they are easily cultured, easily expanded, and repeatedly obtained by simple liposuction under local anesthesia. The number of cells obtained from 1 g of fat yields approximately 5x10^3^ adipose-derived stem cells, which is 500 fold greater than the number of mesenchymal stem cells isolated from 1 g of bone marrow [13, 14]. They also have an increased proliferative ability [15, 16]. The number of ADSCs does not decrease with age, and they are less senescent than bone marrow-derived stem cells [12, 17]. Furthermore, ADSCs are equivalent to, if not better than bone marrow-derived stem cells in terms of their differentiation ability and immunomodulatory effects [18].

ADSCs highly express genes associated with mitosis, inflammation and stress response and usually secrete larger amounts of growth factors and inflammatory cytokines than bone marrow-derived stem cells, which in turn soothes the innate immune response [19, 20]. These cells are also known to have a significant potential for angiogenesis and vasculogenesis [21].

ADSCs have been used for the treatment of several diseases or conditions. Studies using animal models have shown that ADSCs could improve random skin flap survival, promote neovascularization, tissue regeneration and recover left ventricular function of infarcted myocardium [22]. On the other hand, clinical trials using ADSCs have been conducted by different surgeons on cleft lip, breast reconstruction, craniofacial, multiple sclerosis, Crohn’s disease, spinal cord injury and burn renovation [20, 23]. Of note, despite all of the reports concerning the therapeutic role of MSCs and their anti-inflammatory activity, many concerns have been raised about their probable precancerous activity [24, 25].

It has been suggested that telomerase and aldehyde dehydrogenase (ALDH) activity play a role in maintaining the self-renewal, cellular differentiation and expansion capacities of stem cells [26, 27]. An increase in these activities is an indication of cancer. Telomerase activity is regulated by a specific mutation in TP53 and by the expression of some oncogenes and other tumor suppressor genes.

Due to their stemness, plasticity and paracrine effects, ADSCs are a potential therapeutic target for many diseases [20, 23]. In all cases, a large number of cells is essential to generate efficient results, thus the need for culturing and expanding cells in vitro for several weeks. However, long-term cell culture with serial passages may affect stem cell viability, characteristics and differentiation capacity [28–30]. Therefore, a high number of cells at a lower passage number is needed for therapeutic use. The concern is, if the cells are expanded, would they keep their stem cell markers? Would their differentiation and paracrine characteristics be affected? Would these cells acquire cancerous markers, thus having cancerous activity? To answer these above questions, we investigated the potential paracrine activity and stemness of the stromal vascular fraction (SVF) and of minimally-passaged adipose-derived stem cells (ADSCs). In addition, we studied if expanded minimally-passaged cells will promote the expression of cancer markers such as aldehyde dehydrogenase (ALDH) and telomerase activity, and whether minimal serial passages affect the secretome profile of ADSCs.

## Material and Methods

### Isolation of adipose-derived stem cells

The Saint Joseph University Ethics Committee approved the use of specimens obtained by liposuction and approved the entire study. Healthy donors aged 18 to 55 years and undergoing elective liposuction procedures for cosmetic reasons, under local anesthesia provided written informed consent. The stromal vascular fraction (SVF) was separated from the lipoaspirate and 100 to 300 ml were obtained from subcutaneous abdominal, hip and thigh regions. The stromal vascular fraction was separated from the lipoaspirate using a procedure modified by Zuk et al. Briefly, the lipoaspirates were washed with sterile Hank’s Balanced Salt Solution / containing antibiotics, 10000 units /ml penicillin; 10,000 units/ml streptomycin; and 25 mcg/ml amphotericin B (Lonza, Walkersville, MD, USA), and then digested with 0.075% type I collagenase (Sigma-Aldrich, St. Louis, MO, USA) at 37°C for 1–2 h with gentle agitation. The collagenase activity was then neutralized with an equal volume of DMEM-F12 with 10% FBS (Sigma-Aldrich, St. Louis, MO, USA). The samples were centrifuged for 10 min at 600g. The supernatants containing oil, adipocytes and collagenase were discarded, and the pelleted cells i.e. the stromal vascular fraction (SVF), were washed with a PBS/PSA solution (Lonza, Walkersville, MD, USA). Then, it was filtered through a 100 μm filter (BD, Biosciences, San Jose, USA) to remove debris. Finally, the SVF was resuspended in Red Blood Cell Lysis Buffer (Sigma-Aldrich, St. Louis, MO, USA) for 10 min at room temperature. The freshly isolated SVF was transferred to culture flasks containing DMEM-F12 supplemented with 10% FBS (Sigma-Aldrich, St. Louis, MO, USA), and 1% PSA (Lonza, Walkersville, USA) was added to each flask. Cells were then placed into the incubator under at 37°C under 5% CO2, and humidified air. After 48 h, the non-adherent cells were discarded and fresh medium was added. Every 72 h, the culture medium was renewed. The initial passage of the primary cell culture was referred to as passage 0 (P0). The cells were maintained for 21 days. After this time, the cells were harvested by trypsin (0.05%) and cultured in new flasks; this was referred to as passage 1 (P1). The cell viability, cell count number and purity at each passage were evaluated by the Trypan Blue exclusion assay (Sigma-Aldrich, St. Louis, MO, USA) and a hemocytometer. Cells were passaged repeatedly every 21 days until passage 4. The population doubling time (PDT) was calculated using www.doubling-time.com

### Multilineage differentiation and an analysis of adipose-derived stem cells (ADSCs)

ADSCs showed fibroblast-like morphology and differentiation capacity to become adipocytes, osteocytes, and chondrocytes.

#### Adipogenic differentiation

Adipogenic differentiation was induced by MSCGM^TM^ adipogenic medium and MSCGM^TM^ maintenance medium (Lonza, Walkersville, MD, USA) according to the manufacturer’s instructions. Briefly, 2x10^5^ cells were seeded in a 6-well plate and supplemented with 2 ml of MSCGM^TM^. Then the cells were incubated at 37°C with 5% CO2. The medium was changed every 2–3 days until confluency was reached. At 100% confluency, 3 cycles of induction/ maintenance were performed to stimulate optimal adipogenic differentiation. Each cycle consists of feeding the ADSCs with supplemented adipogenic induction medium and culturing for 3 days (37°C, 5% CO2), followed by 1–3 days of culture in supplemented adipogenic maintenance medium. After 3 complete cycles of induction/ maintenance, the cells were cultured for 7 more days in supplemented adipogenic maintenance medium, and the medium was changed every 2–3 days. Then, Oil Red O (Sigma-Aldrich, St. Louis, MO, USA) staining was performed to identify adipose cells.

#### Osteogenic differentiation

Osteogenic differentiation was induced by MSCGM^TM^ osteogenesis medium (Lonza, Walkersville, MD, USA) according to the manufacturer’s instructions. Briefly, 3x10^4^ cells were seeded in a 6-well plate, supplemented with 2 ml MSCGM™, and incubated at 37°C with 5% CO2. After 24 h, osteogenesis was induced by replacing MSCGM^TM^ with osteogenesis induction medium, which was changed every 2–3 days until day 21. Then, Alizarin Red staining (Sigma-Aldrich, St. Louis, MO, USA) was performed to identify osteogenic differentiation.

#### Chondrogenic differentiation

Chondrogenic differentiation was performed as a micromass and three-dimensional pellet culture. Briefly, a micromass was generated by inoculating a droplet of 5 μl of cell suspension (1.6x10^7^cells/ml) in the middle of a well for 2 h in a humidified atmosphere. Then, chondrogenic differentiation was induced using StemPro® Chondrogenesis Differentiation medium (Gibco, Invitrogen, Karlsruhe, Germany). The medium was replaced every 2 to 3 days for 28 days. The micromass was washed with PBS (Lonza, Walkersville, MD, USA) and fixed with a solution of 4% formaldehyde (Sigma-Aldrich, St. Louis, MO, USA). Chondrogenic differentiation was detected using Alcian Blue for 30 min.

In parallel, 5x10^5^ cells were seeded in 15 ml polypropylene culture tubes. The medium was changed every 2–3 days. The chondrogenic pellets were harvested after at least 28 days. Finally, the pellets were fixed with formalin and then embedded in paraffin and stained for glycoproteins with Alcian Blue.

### Flow cytometry analysis

#### Immunophenotyping and cell viability

Immunophenotyping was performed on the SVF and ADSCs cultured from passages 0 through 4 by flow cytometry analysis. The cultured cells were re-suspended at 2.5x10^5^ cells and then incubated with selective fluorochrome-conjugated monoclonal antibodies (mAbs) or with the appropriate isotype antibody controls. After incubation, the cells were washed twice in cold phosphate-buffered saline (PBS) supplemented with 0.5% BSA (Sigma-Aldrich, St. Louis, MO, USA) and fixed with 1% formaldehyde (Sigma-Aldrich, St. Louis, MO, USA) diluted in PBS. The following PE conjugated mAbs were used: anti CD73, anti CD29, anti CD44, anti CD45, anti CD31, anti CD106, anti CD34, anti CD166, anti CD90, and anti CD105 (BD, Biosciences, San Jose, USA). Appropriate isotype controls were used at the same concentrations as the test antibody to determine non specific staining. Furthermore, a viability assay was performed by adding 5 μl of 7-amino actinomycin D (7-AAD) (BD, Biosciences, San Jose, USA) fluorochrome to stain non-viable cells. 10,000 cells were analyzed on a FACSCalibur cell analyzer using the CellQuest software (BD, Biosciences, San Jose, USA). Living cells were identified on the basis of their physical characteristics (forward and side scatter, FSC and SSC, respectively). The results were expressed as a percentage of positive cells.

#### Aldehyde dehydrogenase activity

The aldehyde dehydrogenase activity was evaluated with the ALDEFLUOR^TM^ Kit (Stem Cell Technology, Canada) according to the manufacturer’s instructions. The cellular sample was adjusted to a concentration of 1x10^6^ cells/ml with ALDEFLUOR^TM^ Assay Buffer. The activated ALDEFLUOR^TM^ Reagent, BAAA, which is a fluorescent non-toxic substrate for ALDH, was added. Then, each sample was divided in 2 tubes. The first was labeled “Test”, and the second was labeled “control”, containing the DEAB solution (ALDH inhibitor). The sample was incubated for 30 min at 37°C. Following incubation, all tubes were centrifuged for 5 min at 250 g and the pellets suspended in 0.5 ml ALDEFLUOR^TM^ Assay Buffer. A minimum of 25,000 events were analyzed on a FACSCalibur cell analyzer using the CellQuest software (BD, Biosciences, San Jose, USA). The results were expressed as a percentage of positive cells.

### Telomerase activity measurement

The telomerase activity was assessed in ADSCs. It was measured by real-time PCR using a Quantitative Telomerase Detection Kit (allied Biotech, Inc, USA), which is based on the ability of telomerase presented in cell extracts to synthesize telomeric repeats onto an oligonucleotide substrate, and the resulting extended product is subsequently amplified by PCR. The generated PCR products were then visualized using a highly sensitive DNA fluorochrome, SYBR Green. The detection of the PCR products was measured following the binding of SYBR Green to double-stranded DNA.

ADSC extracts were prepared according to the manufacturer’s protocol. Briefly, the cells were washed twice with cold PBS and then lysed with an appropriate volume of the provided lysis buffer. After a 30 min incubation on ice, the suspension was centrifuged for 30 min at 4°C at 12,000xg. The supernatant was then collected for further telomerase activity and protein determination.

### RNA extraction and PCR

The total RNA was extracted using QIAamp RNA Extraction Kit (Qiagen Inc., Valencia, CA, USA) from ADSCs. The RNA quality and yields were analyzed. The complementary DNA (cDNA) was synthesized from 500 ng of total RNA in a 20 μl reaction using the iScript^TM^ cDNA Synthesis Kit (Bio-Rad Laboratories, CA, USA).

### TP53 expression and sequencing

The expression of TP53 was assessed using (PCR) and specific primer pairs (Table 1). Briefly, 2.5 ng/μl of cDNA was added to 20 μl of the PCR master mix (Solis BioDyne, Estonia). PCR amplification was performed using a Bio-Rad T100™ Thermal Cycler (Bio-Rad Laboratories, CA, USA) and the reactions were subjected to 35 PCR cycles of 95°C for 30 s, 60°C for 40 s, and 72°C for 60 s, followed by a 7-min extension step at 72°C. 5 μl of the PCR products were separated on a 1.5% agarose gel and visualized with SYBR Safe (Invitrogen, UK) staining using the UVP BioDoc system (UVP). The rest of the PCR products were purified using the GenElute^TM^ PCR Clean-Up Kit (Sigma-Aldrich, St. Louis, MO, USA). Sequencing reactions were run on an ABI3500 Genetic Analyzer sequencing system (Applied Biosystems, FosterCity, CA, USA). The DNA sequences were analyzed with ChromasPro v1.22 (Technelysium, Queensland, Australia) and compared to reference sequences using BLAST.

**Table 1:** List of primer sequences.

TP53-1	F -GCGTGCTTTCCACGACG
	R- CCTTCCACTCGGATAAGATG
TP53-2	F- TTGCATTCTGGGACAGCCAA
	R-GGCATCCTTGAGTTCCAAGG
TP53-3	F- CACCATCATCACACTGGAAG
	R-CTGACGCACACCTATTGCAA

### Real-time PCR

Real-time RT-PCR was performed with the SYBR^®^ Green PCR Master Mix (Invitrogen, Carlsbad, CA) in triplicate in a 96-well plate, using an ABI 7500 Real-Time PCR system (Applied Biosystems, Life Technologies, Carlsbad, CA, USA). The reaction conditions were as follows: polymerase activation at 95°C for 10 min, 40 cycles of denaturation at 95°C for 15 s, and annealing and extension at 60°C for 1 min. The relative mRNA expression was normalized against the expression levels of the housekeeper GAPDH using the 2^−ΔΔCT^ method. Each sample was repeated in triplicate for each independent experiment. The primers used are listed in Table 2.

**Table 2:** List of primer sequences for real-time PCR.

	Forward Primer	Reverse Primer
**TP53**	CAAGCAATGGATGATTTGATGCT	TGGGTCTTCAGTGAACCATTGT
**RB**	GCAAATTGGAAAGGACATGTGA	GAAACTTTTAGCACCAATGCAGAA
**c-Myc**	CACCACCAGCAGCGACTCT	TTCCACAGAAACAACATCGATTTC
**MDM2**	ATATACCATGATCTACAGGAACTTGGTAGT	GGTGACACCTGTTCTCACTCACA
**hTERT**	CGTCCAGACTCCGCTTCATC	GACGTAGTCCATGTTCACAATCG

### Cytokine levels

The levels of PGE_2_, Il-6, TIMP1, TIMP2, and VEGF were measured in cell supernatant using enzyme-linked immunoabsorbent assays (ELISA-R&D Abingdon, United Kingdom) according to the manufacturer’s protocol without any modification. The sensitivity of the technique was 0.7 pg/ml, 0.08 ng/ml, 0.06 ng/ml, 0.041 ng/ml, and 9 pg/ml respectively. The level of STC1was measured using an ELISA kit from antibodies-online (GmbH Schloss-Rahe-Str. 52072 Aachen Germany). The kit’s sensitivity is 78 pg/ml. For the ELISAs, all assays were performed in duplicate.

### Statistical analysis

The statistical analyses were performed using Graph Pad Prism 5.0. The values are presented as the mean ± standard error mean of the mean (SEM). For the data from each type of experiment, the most suitable statistical analysis was conducted: Student’s t‐test, and Wilcoxon test for qPCR analysis. Differences were considered significant at P < 0.05.

## Results

### Morphology and immunophenotyping

**Fig 1:**
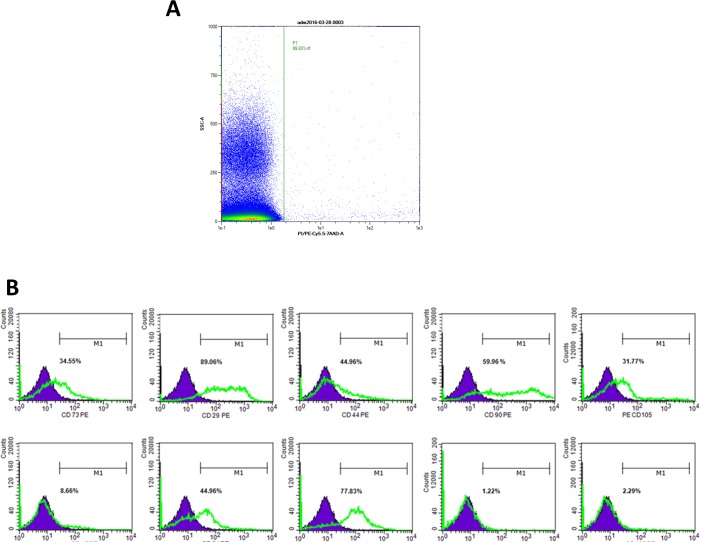
Immunophenotype and viability of the stromal vascular fraction (SVF). The cell viability was confirmed by flow cytometry analysis. (A) The viability of the stromal vascular fraction cells. (B) The flow cytometry histograms of the SVF for a representative donor are displayed. The percentage of cells that stained positive is indicated in the upper right corner of each panel. The green line indicates the positively stained cells, whereas the purple line indicates the isotype-matched monoclonal antibody control.

**Fig 2:**
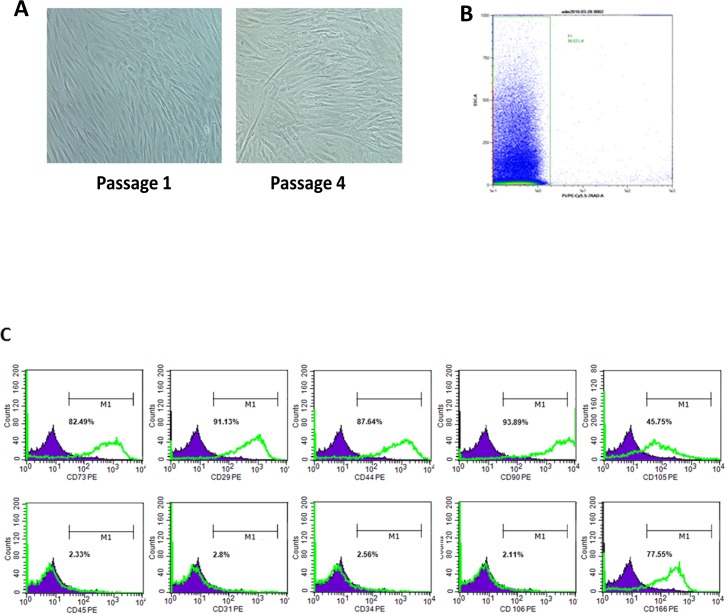
Morphology, immunophenotype and viability of adipose-derived cells. The morphology, viability and immunophenotype of adipose-derived cells during passaging. ADSCs have fibroblastic features at P1, and P4. (A) The cell viability was confirmed by flow cytometry analysis. (B) The viability of ADSCs. (C) The flow cytometry histograms for a representative donor are displayed at passage 4 (P4). The percentage of cells stained positive is indicated in the upper right corner of each panel. The green line indicates the positively stained cells, whereas the purple line indicates the isotype-matched monoclonal antibody control.

To characterize our primary cells and cells at different passages (P0-P4), flow cytometry analysis was performed on the stromal vascular fraction (SVF) and passaged ADSCs. The stromal vascular fraction (SVF) was successfully isolated from the lipoaspirates of 6 patients. Flow cytometry analysis showed that cell viability was 95% ± 4 (Fig 1A), and the cells were positive for CD73 (33.62% ± SEM 6.27), CD29 (69.53% ± SEM 8.61), CD44 (48.52% ± SEM 5.94), CD90 (50.52% ± SEM 6.66), CD105 (21.09% ± SEM 3.02), CD34 (55.09% ± SEM 9.64), and CD31 (32.95% ± SEM 3.99); slightly positive for CD45 (11.56% ± SEM 2.1) and negative for CD106 (1.51% ± SEM 0.41) and CD166 (2.39% ± SEM 0.76) (Fig 1B). The seeded cells were purified by changing the media and subculturing to become ADSCs, which were adherent to the plastic and showed a fibroblast-like morphology (Fig 2A). After 21 days of culture, the cells became more homogenous; the viability was not affected at 95% ± 4 (Fig 2B), nor their stemness as shown by their stem cells markers and differentiation capacity (Table 3). Flow cytometry analysis at P4 showed an increase in the level of expression of the positive markers CD73 (82.02% ± SEM 4.84), CD29 (90.28% ± SEM 2.24) CD44 (88.3% ± SEM 1.78), CD90 (93.19% ± SEM 1.65), CD105 (59.63% ± SEM 8.13), and in contrast to the total lack of expression of the endothelial and hematopoietic markers CD34 (2.17% ± SEM 0.34), CD 31 (2.27% ± SEM 0.43), CD45 (2.18% ± SEM 0.31), and CD106 (2.31% ± SEM 0.11). CD166 became positive with increasing passages (43.37% ± SEM 11.94) at P4 (Fig 2C).

**Table 3:** Cell yield and the population doubling time (PDT) of passaged ADSCs (n = 6).

	Passage 1	Passage 2	Passage 3	Passage 4
Initial cell number (x10^6^)	11.25 ± 0.72	11.25 ± 1.25	10.75 ± 0.75	10 ± 0.5
Final cell number (x10^6^)	48.63 ± 3.74	47.13 ± 11.67	47.38 ± 14.54	34.13 ± 5.309
Doubling time	9.989 ± 0.29	11.62 ± 1.783	11.75 ± 1.92	12.55 ± 1.184
Viability	95 ± 4%	95 ±4%	95 ±4%	95 ± 4%

### Multilineage differentiation

**Fig 3:**
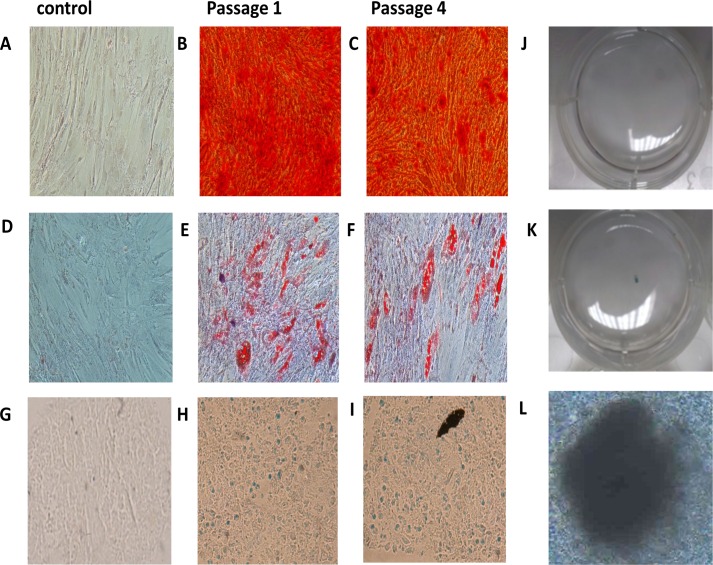
Differentiation capacity of adipose-derived cells. Cells at P1 and P4 were incubated for three weeks with adipogenic, osteogenic, and chondrogenic media. Representative images (B, C) of the intracellular lipid droplets that were confirmed by Oil Red O staining compared to (A) the control cells. (E, F) The presence of calcium deposit was visualized by Alizarin Red staining compared (D) to the control. (H, I) The presence of GAG was confirmed with Alcian Blue staining and (K, L) the solid chondrogenic micromass compared to the control (G, J).

To test the differentiation potential of ADSCs after consecutive passaging to reach P4, a classic mesoderm differentiation protocol was implemented. We first observed that cells at P1-P4 retained their ability to differentiate into adipocytes, osteocytes and chondrocytes. Lipid droplets staining with Oil Red O had a red orange color. Undifferentiated ADSCs do not contain calcium, whereas ADSCs differentiated into osteoblasts showed an orange-red color when stained with Alizarin Red, an indicator of the presence of calcium. ADSCs differentiated into chondrocyte-like cells as visualized by Alcian blue staining aggrecan, which is an indicator of chondrogenic differentiation (Fig 3).

### Aldehyde dehydrogenase (ALDH) activity

Telomerase and ALDH activity are increased in cancerous states and can be used as cancer markers. To prove that passaged ADSCs do not acquire a tumorigenic phenotype, we looked at the expression of ALDH and telomerase activity.

**Fig 4:**
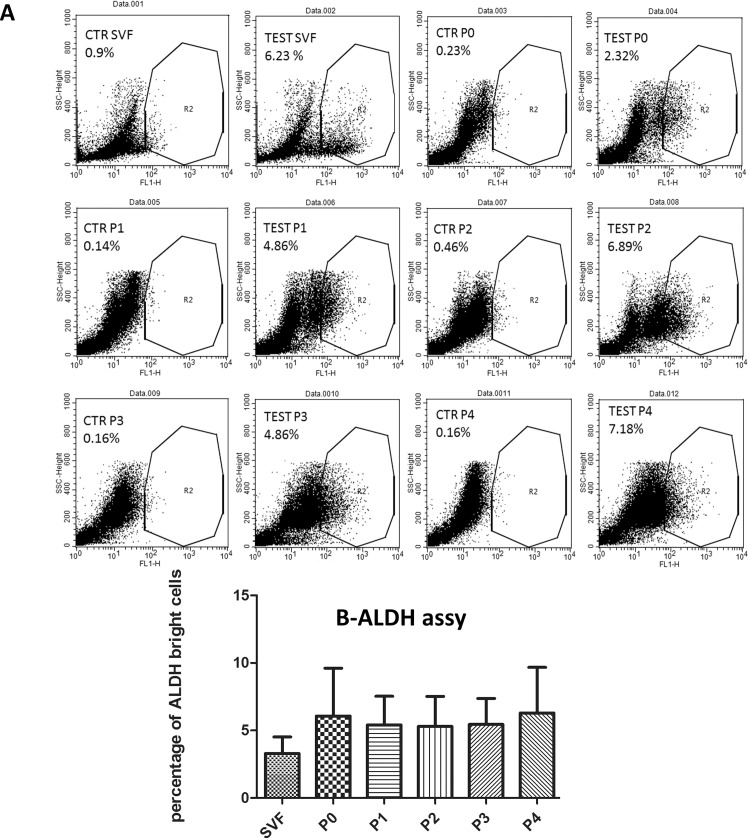
Aldehyde dehydrogenase activity. One million cells were separated in two tubes; 5 μl of a 1.5 mM DEAB stock solution was added to the “control” and 5 μl of activated ALDEFLUOR^®^ Substrate per mL of sample was added to the “test” tube. Cells were then incubated for 30 min in a 37°C. Finally, the tubes were analyzed by a flow cytometer. (A) A representative flow cytometry analysis of the ALDH activity; and (B) the average of ALDHbr cells results from 10 donors. The data represent the mean + SEM.

Based on flow cytometry analysis, the percentage of SVF derived from 6 patients showed on average 3.925% ±1.196 ALDH^br^ cells, with a slightly yet non-significant increase through passages P0-P4, with the average value being 6.1%. This population was not observed in the presence of diethylaminobenzaldehyde, a potent inhibitor of ALDH (Fig 4A and 4B).

### Telomerase activity throughout the passages

**Fig 5:**
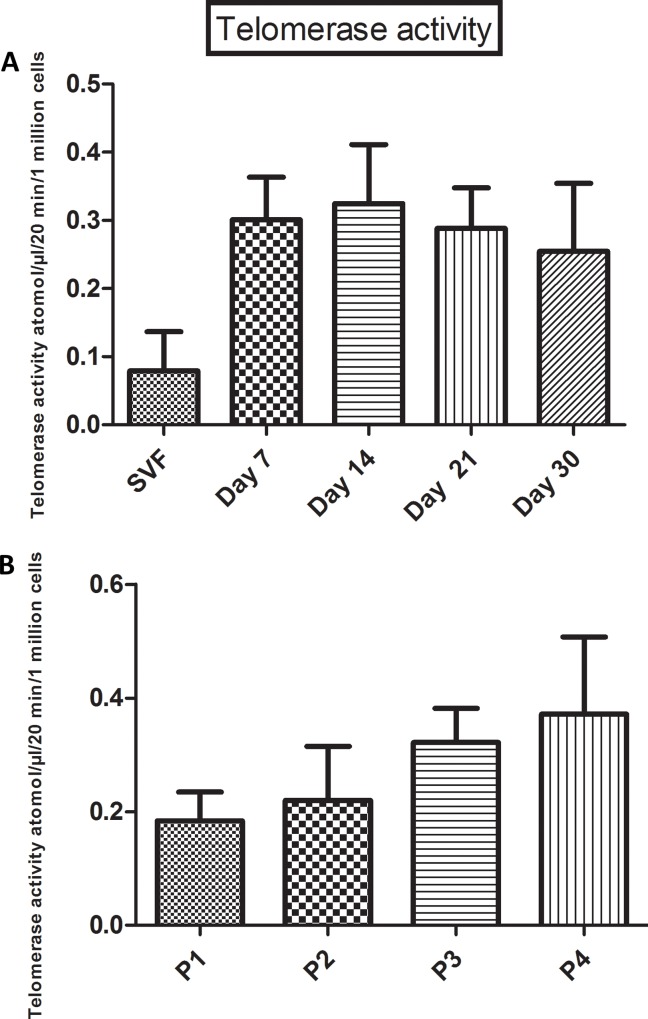
Telomerase activity. A comparison of the relative telomerase activities for (A) SVF and ADSCs at a specific time and date (7,14,21 and 30 days) and (B) through the passages (P1-P4). One million cells were re-suspended in 1×Lysis Buffer, and then incubated on ice for 30 min. The telomerase activity was detected by qPCR as indicated, in the Materials and Methods section. The results are from 10 donors. The data represent the mean ± SEM.

Telomerase activity was non-significantly increased in both SVF and ADSCs. During incubation from day 7 to day 30 (Fig 5A), the telomerase activity was shown to be stable, with values of 0.301±0.062, and 0.275 ±0.118, respectively. During the passages, the telomerase activity did not increase significantly (Fig 5B).

### TP53 mutation detection

**Fig 6:**
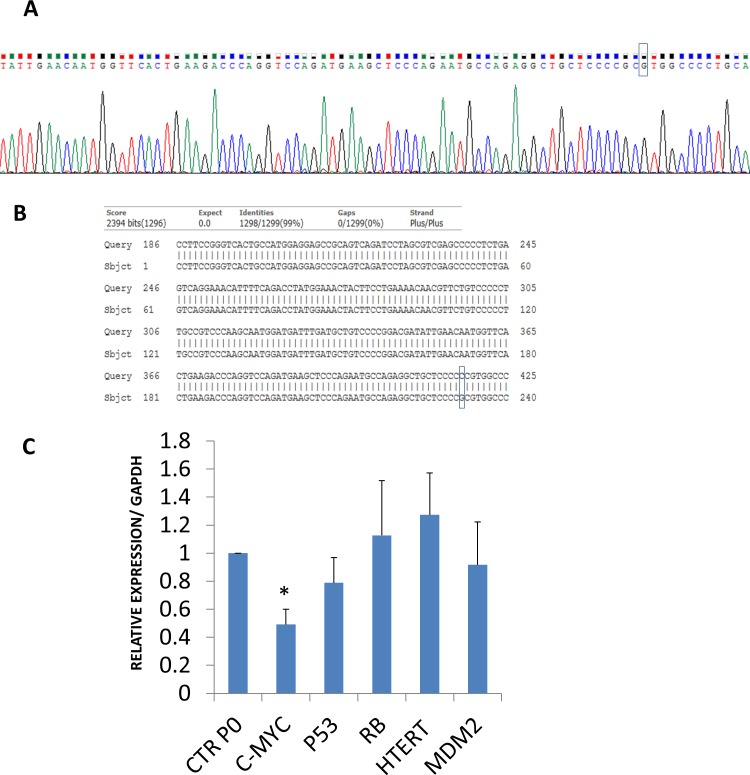
TP53 sequencing and the relative expression of the tumor suppressor genes TP53 and RB and the oncogenes c-Myc, MDM2, and hTERT. (A) The PCR products of TP53 exon 2 to 11 from passage 0 and 4 ADSCs. (B) BLAST (Basic Local Alignment Search Tool) was used to compare the TP53 sequence to the wild type TP53 sequence. This figure from one donor is a representative for all donors. (C) Relative expression of the tumor suppressor genes and oncogenes. Total RNA was purified and the cDNA was prepared from total RNA using an iScript kit. The relative expression levels of the tumor suppressor genes TP53 and RB, and the expression levels of the oncogenes c-Myc, MDM2, and hTERT from ADSCs at P0 and P4 was performed using quantitative PCR with specific primers as indicated in the Material and Methods section. The results are the mean from 5 donors. The data were normalized to GAPDH and presented as the mean ± SEM. An * indicates a p value of < 0.05.

TP53 mutations play an important role in tumorigenesis. To demonstrate that passaged ADSCs did not acquire any mutation in TP53 during passages (P0-P4), we performed a sequencing of TP53 exon 2 to 11 from ADSCs at passages 0 and 4. The PCR products were sequenced and BLAST was used to compare the TP53 sequence obtained to the wild type TP53. It showed a single nucleotide polymorphism compared to the wild type at nucleotide 417 in which C is substituted with G (Fig 6A).

### Tumor suppressor gene and oncogene levels of ADSCs through passages

The analysis of the tumor suppressor genes TP53 and RB and the oncogene MDM2 showed that the ADSCs expressed these genes at steady levels throughout the passages. In contrast, the relative expression of c-Myc decreased significantly at P4 0.4918 ±0.10904 (p = 0.04) compared to P0. The relative expression of hTERT was not detected before 35 cycles. After that, a slight expression was detected, but it remained unchanged during the passages (Fig 6B).

### Paracrine factors

**Fig 7:**
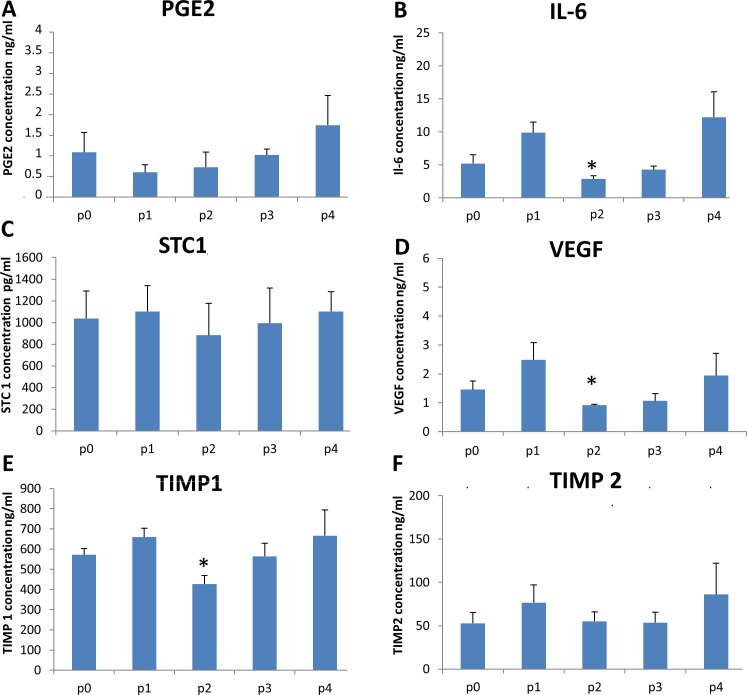
The paracrine activity of ADSCs during passaging. The levels of immunomodulatory proteins (A) PGE_2_ and (B) IL-6; (C) the anti-apoptotic protein STC1; and the trophic proteins (D) VEGF, (E) TIMP1, and (F) TIMP2 released into the culture medium were measured by an ELISA assays as described in the Material and Methods section. The results are the mean from 7 donors. The data represent the mean ± SEM. An * indicates a p value of < 0.05.

To investigate the unchanged properties of ADSCs during passaging, we evaluated the expression of the paracrine factors secreted by stem cells. As illustrated in Fig 7, the levels of PGE_2_, STC1, and TIMP2 did not change, but the levels of IL-6, VEGF, and TIMP 1 decreased significantly at P2. The protein expression of TSG6 was not detected; however, the mRNA level was increased 24 ± 7.56 fold from P0 to P4 (p = 0.043) (Fig 7).

## Discussion

Adipose-derived stem cells are used more often in clinical therapy. A large number of cells is essential to generate efficient results, with the need for culturing and expanding the cells in vitro for several weeks. However, long-term cell culture with serial passages may affect stem cell characteristics and the differentiation capacity of these cells [28–30]. The aim of this study was to expand adipose-derived stem cells, while minimizing the number of passages to achieve a higher cell number without losing the stemness, viability and differentiation capacity of ADSCs. We see this as the novelty of our study. We started our work by comparing the morphology, number, and viability of cells passaged after 7, 14, 21 and 30 days (S1 Fig). Preliminary assays were performed and we established that the highest number of cells is obtained when the cells were passaged at 21 days, cultured, and then passaged again until P4. We also demonstrated that the adipose-derived stem cells retain their morphologic, phenotypic, and plasticity characteristics over the passages. Our results showed that the cells maintained in culture for 12 weeks possessed stable telomerase and ALDH activities, and did not have a TP53 mutation. Moreover, the relative expression levels of TP53, RB, and MDM2 were not affected. The relative expression of c-Myc decreased, and that of hTERT was not detected. The relative expression of TSG6, which is an immunomodulatory factor, increased 24-fold throughout the passages. Finally, the levels of PGE_2_, STC1, and TIMP2 did not change but the levels of IL-6, VEGF, and TIMP 1 decreased at P2.

The expanded ADSCs showed adherence to plastic with a fibroblast-like morphology while forming a homogenous population with the positive stromal stem cells markers CD 73, CD 29, CD44, CD90, CD105, and CD 166. These cells were also negative for CD 106 (VCAM), CD 45, CD31 and CD 34. It was important to demonstrate that these cells not only keep their stem cells markers after passaging but also retain their capacity to differentiate. This was demonstrated by the differentiation of ADSCs at P4 into adipocytes, osteocytes, and chondrocytes.

After verifying the capacity of stem cells to differentiate, it was crucial to show that these cells do not express an increase in cancer markers, such as aldehyde dehydrogenase (ALDH), and telomerase activity. In fact, ALDH can be effectively used as a cancer stem cell marker in tissue types that normally do not express ALDH at high levels, such as breast and lungs [31].

The ALDH-bright subset in human cancer cells can be determined using the criteria of the sorting gates. The population in the top 10–20% have been considered ALDH high, and the population in the bottom 10–20% can be considered ALDH low [32].

Cells that highly express ALDH are highly tumorigenic and have enhanced stem cells characteristics in vitro and in vivo compared to cells with low ALDH1 activity. Our results showed that the average number of ALDH^bri^ cells in the SVF was 3.925% and reached 6% during passaging which mean that the percentage of ALDH^bri^ cells did not change significantly during the passages confirming the finding of Estes et al. who found the average percentage of ALDH^bri^ cells at P2 was 2.32% [33]. This is in contrast with Mitchell et al., who found that ALDH^bri^ cells reached 70% between passages P0 to P4 and then decreased to 10% at P9 [34].

A second predictor of cancerous status is the increase in telomerase activity in cells [35]. It was reported that telomerase activity in human bone marrow-derived stem cells is higher than stem cells derived from adipose tissue [36] and that the telomerase activity will decrease markedly in the two stem cells sources over extended culture [17]. In our study, we showed that the SVF expresses a low level of telomerase activity, which was also stable during extended culture from day 7 to day 30. After passaging the cells to P4, the telomerase activity increased insignificantly during passages from P0 to P4. Similar results were reported by W. S. Wan Kamarul Zaman et al. [37]. However, other studies failed to detect the telomerase activity in adipose-derived stem cells and in bone marrow stem cells [38, 39]. The discrepancies found between our data and the data of others in telomerase changes might be due to the kits and methods used to detect the telomerase activity. In fact, the kit used in our study is very sensitive and can detect the telomerase activity in trace amount of sample, such as 1–5 cells in 293T cells (quantitative telomerase detection kit Allied Biotech, Inc 10075 Tyler Place, Suite 19, Ijamsville, MD 21754).

To further investigate the non-cancerous activity of our passaged cells, we looked at the expression of various genes that are implicated in the regulation of telomerase activity directly or indirectly. These genes included three oncogenes hTERT, c-Myc, and MDM2, and two tumor suppressor genes TP53 and RB [40, 41]. The hTERT protein is the catalytic subunit of the protein complex and has reverse transcriptase activity. The deregulation of hTERT is a key factor in carcinogenesis[42]. In our study, the expression of hTERT was not detected by RT-PCR but had low expression, as indicated by qPCR (after 35 cycles). Its expression was stable during the passages, which induces low telomerase activity, confirming the safety of passaging. Similar results were reported by other studies, such as those by Nakamura et al. 1997 [43], which reported the presence of hTERT transcripts in somatic cells. W. S. Wan Kamarul Zaman et al. 2012 [37] remarked on the presence of the hTERT transcript in low levels in adipose-derived stem cells [44]. However, in other studies, the authors have reported the absence of hTERT expression [38, 45].

c-Myc is considered an oncogene, and its overexpression affects normal cell function and leads to the spontaneous transformation of ADSCs [46]. It was shown that the expression of c-Myc in human ADSCs decrease with increased passaging [47]. For the porcine adipose and bone marrow-derived stem cells, the relative expression of c-Myc was not affected by passaging [48]

In our study, we observed that the relative expression of c-Myc decreased with passaging, confirming the normal status of adipose-derived stem cells.

MDM2 is another oncogene shown to be overexpressed in a variety of human tumors, more specifically in soft tissue sarcomas [49, 50]. To our knowledge, we are the first laboratory to study the expression of MDM2 during passaging. We found that the relative expression of MDM2 was stable during passaging.

TP53 and RB are the main tumor suppressor genes. They regulate telomerase activity and maintain cell hemostasis by controlling cell cycle, apoptosis and senescence. Alterations in these genes are frequently reported in liposarcomas and sarcomas [39, 51]. TP53, the “guardian of the genome”, is not only a tumor suppressor gene that requires a TP53-specific mutation for tumorigenesis, but it is also involved in the proliferation and differentiation of mesenchymal stem cells [52]. Some studies have reported a correlation between a TP53 specific mutation and telomerase activity [53]. Rubio et al. showed that a TP53 mutation is sufficient to transform adipose-derived stem cells or bone marrow-derived stem cells and lead to a sarcoma phenotype [54, 55]. Thus, to investigate if our passaged cells show a TP53 mutation, we expanded and sequenced the cells. No-mutation was observed from exon 2 to exon 11, and only a Pro/Arg polymorphism at amino acid residue 72 was detected. The relative expression levels of TP53 and RB were not affected by passaging for our ADSCs. This led us to conclude that our ADSCs not only did not manifest an increase in oncogene expression, but also maintained a stable level of the suppressor gene, excluding any malignant transformation during passaging. The results of the ALDH and telomerase activity of ADSCs obtained in our study endorse the safety of passaging the cells and suggest a safety profile for proliferation and that the number of cells could be increased for therapeutic purposes.

To confirm our findings of the unchanged properties of ADSCs during passaging, we compared the secretion of the stem cells from P0 to P4. To our knowledge, this is the first work that evaluates the paracrine factors secreted by ADSCs during passaging. We have shown that (PGE_2_), stanniocalcine1 (STC1), and tissue inhibitors metalloproteinases2 (TIMP2) were not affected during passaging whereas, interleukin 6 (IL-6), vascular endothelial growth factor (VEGF), and tissue inhibitors metalloproteinases1 (TIMP1) decreased significantly at P2. Considering the role of each cytokine, this finding indicates that our cells are immunomodulatory.

PGE_2_ is considered the main soluble immunosuppressant protein secreted by MSCs [56]. Studies have reported that the immunosuppressive role of ADSCs is mediated by the secretion of PGE_2_ [57, 58]. It is responsible for switching the immune response from a type 1 (Th1) to type 2 (Th2) [59]. Our study showed that the levels of PGE_2_ were not affected during passaging. IL-6 contributes to the immunosuppressive properties of MSCs. It stimulates the secretion of IL-10 via immature dendritic cells; it triggers macrophages to decrease TNF alpha secretion, and promotes apoptosis among neutrophils [60–62]. The level of IL-6 secretion remained unchanged through passaging; however, it was significantly decreased at P2. We are the first laboratory to identify this decrease at P2, and further experiments are underway to determine the reason behind it. TSG6 is a multifunctional protein with anti-inflammatory properties that plays an important role in tissue remodeling [63, 64]. After 24 h of culturing ADSCs, the level of TSG-6 increased 25 fold from P0 to P4. However, we were not able to detect its production using an ELISA. In fact, according to Lee et al., the cells need to be cultured for a longer period than 24 h to express it [65]. This could be one of the explanations why TSG-6 was not detected in the supernatant of our cells.

To our knowledge we are the first to investigate the STC1 levels secreted by monolayer cultured cells. To evaluate the paracrine effects of ADSCs, we studied their secretion of STC1, TIMP 1 and 2, and VEGF. The expression of stanniocalcin 1 (STC1) was stable during passaging which shows that the ADSC’s anti- inflammatory and anti-apoptotic potency mediated by STC1 [66, 67] is not affected by passaging.

Tissue inhibitors of metalloproteinases (TIMPs) 1 and 2 are multifunctional proteins that maintain tissue remodeling in the extracellular matrix and contribute to angiogenesis, cell growth, and anti-apoptotic activity [68–74]. VEGF is a pro-angiogenic and anti -apoptotic protein [75]. The expression levels of both TIMP 1 and 2 and VEGF remained the same throughout all passages, despite a significant decrease in TIMP1 and VEGF at P2. The stability of the levels of TIMP 1and 2 and VEGF during passaging is of key importance because it has been suggested that the overexpression of TIMP1 and VEGF is associated with oncogenic cellular transformation. Furthermore, the simultaneous decrease in the expression of TIMP1, IL-6, and VEGF at P2 could be explained by the fact that a correlation exists among these specific markers as suggested by Ihn et al [76]. Experiments are being conducted to further explore the relationship among them.

## Conclusion

Keeping ADSCs in culture for 12 weeks following our established protocol will yield the number of cells needed for therapy while maintaining their stemness, differentiation capacity and paracrine secretions. This is achieved without conferring a cancerous state or capacity. An in vivo study is currently being conducted in our lab to confirm the immunomodulation and non-tumorigenic activities of ADSCs.

## Supporting Information

S1 Fig Cell number at 0, 7, 14 and 21 days. ADSCs were cultured for 0, 7, 14, 21, and 30 days. Cell count by Trypan Blue showed that the number of cells increased significantly at day 21 then decreased at day 30. We have chosen day 21 as our optimal culture day. The results are the mean from 6 donors. The data represent the mean ± SEM. An **indicates a p value of < 0.01 and *** < 0.001.
(TIF)
