# Supplementary figures and images for: An Evaluation of the Stemness, Paracrine, and Tumorigenic Characteristics of Highly Expanded, Minimally Passaged Adipose-Derived Stem Cells
Source: PLoS One. 2016 Sep 15;11(9):e0162332. doi: 10.1371/journal.pone.0162332 (PMC5024991; doi:10.1371/journal.pone.0162332)

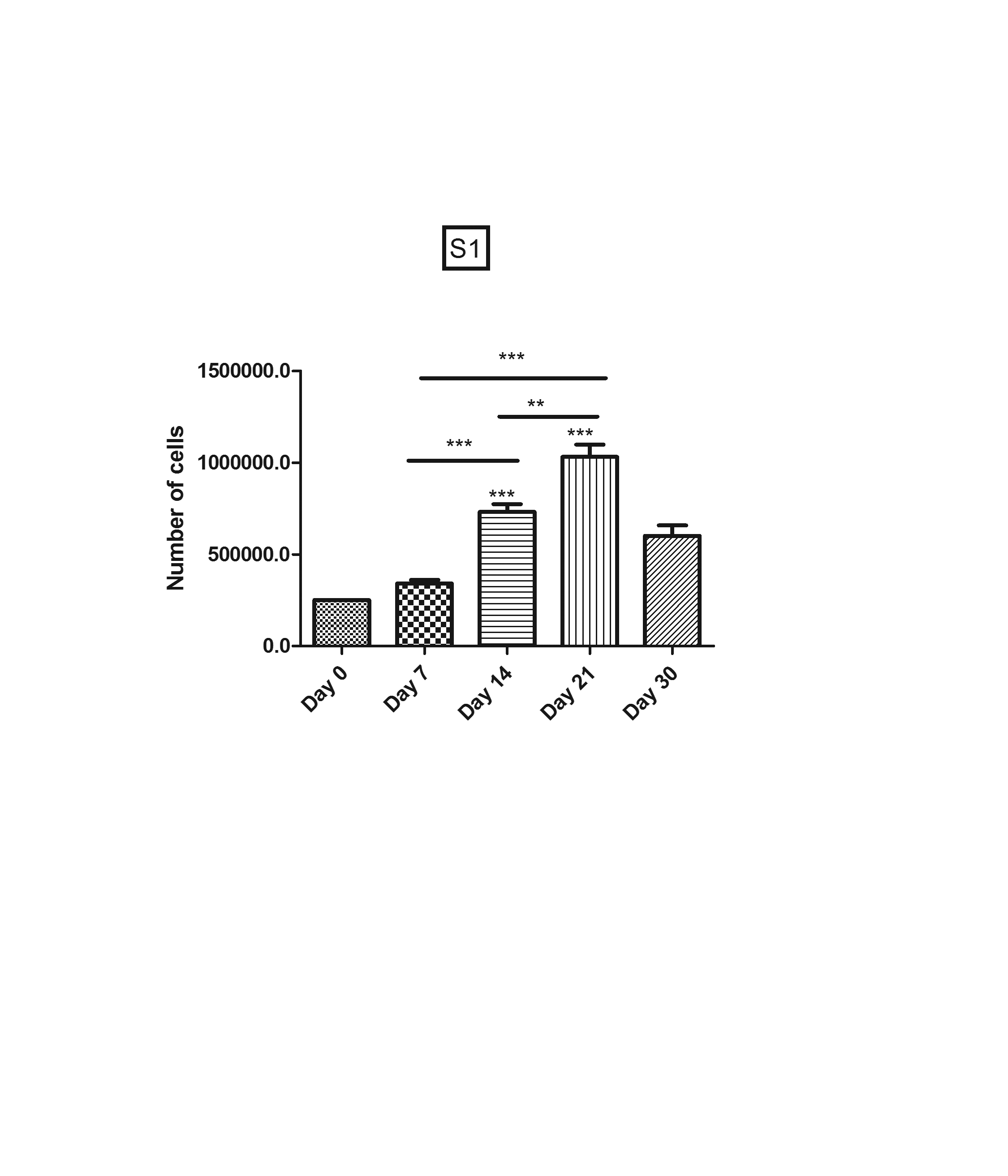

Supplement: S1 Fig — ADSCs were cultured for 0, 7, 14, 21, and 30 days. Cell count by Trypan Blue showed that the number of cells increased significantly at day 21 then decreased at day 30. We have chosen day 21 as our optimal culture day. The results are the mean from 6 donors. The data represent the mean ± SEM. An **indicates a p value of < 0.01 and *** < 0.001. (TIF) [file pone.0162332.s001.tif]
